# Supplementary material for: Oncologic outcomes of Bacillus Calmette-Guérin therapy in elderly patients with non-muscle-invasive bladder cancer: A meta-analysis
Source: PLoS One. 2022 May 19;17(5):e0267934. doi: 10.1371/journal.pone.0267934 (PMC9119482; doi:10.1371/journal.pone.0267934)
Supplement: S1 File — (DOCX) [file pone.0267934.s001.docx]

**PubMed**

("Age Factors"[MeSH Terms] OR "age"[All Fields]) AND ("BCG Vaccine"[MeSH Terms] OR "BCG"[All Fields] OR "bacillus calmette guerin"[All Fields] OR "Calmette Guerin Bacillus"[All Fields] OR "bacillus calmette guerin"[All Fields]) AND ("Treatment Outcome"[MeSH Terms] OR "Treatment Effectiveness"[All Fields] OR "Treatment Efficacy"[All Fields] OR "Clinical Efficacy"[All Fields]) AND ("Urinary Bladder Neoplasms"[MeSH Terms] OR "Bladder Tumor"[All Fields] OR "Urinary Bladder Neoplasms"[All Fields] OR "Bladder Cancer"[All Fields] OR "Cancer of Bladder"[All Fields] OR "Bladder Tumor"[All Fields] OR "carcinoma in situ"[All Fields]) 108 results

Google scholar

( TITLE-ABS-KEY ( *"BCG Vaccine"*  OR  *"BCG"*  OR  *"bacillus calmette guerin"*  OR  *"Calmette Guerin Bacillus"*  OR  *"bacillus calmette guerin"* ) )  AND  ( TITLE-ABS-KEY ( *"aged"*  OR  *"aged"*  OR  *"elderly"* ) )  AND  ( TITLE-ABS-KEY ( *"Urinary Bladder Neoplasms"*  OR  *"Bladder Tumor"*  OR  *"Urinary Bladder Neoplasms"*  OR  *"Bladder Cancer"*  OR  *"Cancer of Bladder"*  OR  *"Bladder Tumor"*  OR  *"neoplasm"*  OR  *"carcinoma in situ"*  OR  *"carcinoma"* ) )  AND  ( TITLE-ABS-KEY ( *"Treatment Outcome"*  OR  *"Clinical Efficacy"*  OR  *"Treatment Effectiveness"*  OR  *"Treatment Efficacy"*  OR  *"efficacy"* ) )  1092 results

**embase**

| **1** | ('bcg vaccine'/mj OR 'bcg':ti,ab,kw OR 'calmette guerin bacillus':ti,ab,kw OR 'bacillus calmette guerin':ti,ab,kw OR 'bcg vaccine':ti,ab,kw) AND [1998-2021]/py | **50676** |
| --- | --- | --- |
| **2** | ('treatment outcome'/mj OR 'efficacy'/mj OR 'treatment effectiveness':ti,ab,kw OR 'therapy':ti,ab,kw OR 'clinical efficacy':ti,ab,kw) | **3029949** |
| **3** | ('bladder tumor'/mj OR 'urinary bladder neoplasms'/mj OR 'urinary bladder neoplasms':ti,ab,kw OR 'bladder cancer':ti,ab,kw OR 'bladder tumor':ti,ab,kw OR 'carcinoma in situ':ti,ab,kw OR 'cancer of bladder':ti,ab,kw) | **90922** |
| **4** | 'age'/mj OR 'aged'/mj OR 'age':ti,ab,kw OR 'aged':ti,ab,kw OR 'elderly':ti,ab,kw | **4564917** |
| **5** | **#1 AND #2 AND #3 AND #4** | **563** |

**Web of science**

| **1** | AB=(BCG  OR  bacillus  calmette  guerin  OR  Calmette  Guerin  Bacillus  OR  bacillus  calmette  guerin)  OR  TI=(BCG  OR  bacillus  calmette  guerin  OR  Calmette  Guerin  Bacillus  OR  bacillus  calmette  guerin)  OR  AK=(BCG  OR  bacillus  calmette  guerin  OR  Calmette  Guerin  Bacillus  OR  bacillus  calmette  guerin)  Indexes=SCI-EXPANDED, SSCI, A&HCI, CPCI-S, CPCI-SSH, BKCI-S, BKCI-SSH, ESCI, CCR-EXPANDED, IC Timespan=All years | **22,665** |
| --- | --- | --- |
| **2** | AB=(Urinary  Bladder  Neoplasms  OR  Bladder  Tumor  OR  Urinary  Bladder  Neoplasms  OR  Bladder  Cancer  OR  Cancer  of  Bladder  OR  Bladder  Tumor  OR  carcinoma  in  situ)  OR  TI=(Urinary  Bladder  Neoplasms  OR  Bladder  Tumor  OR  Urinary  Bladder  Neoplasms  OR  Bladder  Cancer  OR  Cancer  of  Bladder  OR  Bladder  Tumor  OR  carcinoma  in  situ)  OR  AK=(Urinary  Bladder  Neoplasms  OR  Bladder  Tumor  OR  Urinary  Bladder  Neoplasms  OR  Bladder  Cancer  OR  Cancer  of  Bladder  OR  Bladder  Tumor  OR  carcinoma  in  situ)  Indexes=SCI-EXPANDED, SSCI, A&HCI, CPCI-S, CPCI-SSH, BKCI-S, BKCI-SSH, ESCI, CCR-EXPANDED, IC Timespan=All years | **93,019** |
| **3** | AB=(age  OR  aged  OR  elderly)  OR  TI=(age  OR  aged  OR  elderly)  OR  AK=(age  OR  aged  OR  elderly)  Indexes=SCI-EXPANDED, SSCI, A&HCI, CPCI-S, CPCI-SSH, BKCI-S, BKCI-SSH, ESCI, CCR-EXPANDED, IC Timespan=All years | **3,539,009** |
| **4** | AB=(Treatment  Outcome  OR  Treatment  Effectiveness  OR  Treatment  Efficacy  OR  Clinical  Efficacy  OR  efficacy)  OR  TI=(Treatment  Outcome  OR  Treatment  Effectiveness  OR  Treatment  Efficacy  OR  Clinical  Efficacy  OR  efficacy)  OR  AK=(Treatment  Outcome  OR  Treatment  Effectiveness  OR  Treatment  Efficacy  OR  Clinical  Efficacy  OR  efficacy)  Indexes=SCI-EXPANDED, SSCI, A&HCI, CPCI-S, CPCI-SSH, BKCI-S, BKCI-SSH, ESCI, CCR-EXPANDED, IC Timespan=All years | **1,623,441** |
| **5** | **#1 AND #2 AND #3 AND #4** | **114** |
